# Construction of a Food Safety Evaluation System Based on the Factor Analysis of Mixed Data Method

**DOI:** 10.3390/foods13172680

**Published:** 2024-08-25

**Authors:** Yiqiong Liu, Shengmei Cai, Xuelei He, Xiaowei He, Tianli Yue

**Affiliations:** 1 College of Food Science and Technology, Northwest University, Xi’an 710069, China; liuyiqiong@stumail.nwu.edu.cn; 2 School of Information Sciences and Technology, Northwest University, Xi’an 710069, China; 3 Laboratory of Nutritional and Healthy Food-Individuation Manufacturing Engineering, Xi’an 710069, China; 4 Research Center of Food Safety Risk Assessment and Control, Xi’an 710069, China

**Keywords:** food safety, evaluation index, sampling inspection, expert elicitation, machine learning, FADM

## Abstract

Food safety evaluation, which aims to reflect food safety status, is an important part of food safety management. Traditional food evaluation methods often consider limited data, and the evaluation process is subjective, time-consuming, and difficult to popularize. We developed a new food safety evaluation system that incorporates simple qualification degrees, food consumption, project hazard degrees, sales channels, food production regions, and other information obtained from food safety sampling and inspection to reflect the food safety situation accurately, objectively, and comprehensively. This evaluation model combined the statistical method and the machine learning method. The optimal distance method was used to calculate the basic qualification degree, and then expert elicitation via a questionnaire and the factor analysis of mixed data method (FADM) was applied to modify the basic qualification degree so as to obtain the food safety index, which indicates food safety status. Then, the effectiveness of this new method was verified by calculating and analyzing of the food safety index in region X. The results show that this model can clearly distinguish food safety levels in different cities and food categories and identify food safety trends in different years. Thus, this food safety evaluation system based on the FADM quantifies the real food safety level, screens out cities and food categories with high food safety risks, and, finally, helps to optimize the allocation of regulatory resources and provide technical and theoretical support for government decision-making.

## 1. Introduction

In recent years, the issue of food safety has become an urgent problem that is tightly associated with national economic development and social stability. With economic and social development, the food industry has grown rapidly and become an important pillar industry of the national economy [1]. Governments around the world continue to strengthen food safety supervision to ensure food safety for building a healthy society and improving people’s well-being [2,3]. Although the food safety situation is generally stable and improving, food safety accidents occur from time to time. From the “melamine milk powder” incident in 2008 to the “gutter oil hotpot” incident in 2016 and the “fake Thai rice” incident in 2023, the exposure of each food safety issue has aroused widespread concern in the community. In order to further improve food safety management, food safety evaluation has been proposed and promoted. Food safety evaluation refers to describing food safety status in one region or within a certain time by processing food safety-related information [4]. The evaluation system of food safety is an important part of food safety management, which can reflect food safety status comprehensively from data on food harmful substance detection, production, and distribution [5]. The research on food safety evaluation not only contributes to the development of food safety management but also provides theoretic and scientific support for government decision-making [6]. In China, the government has accumulated a large amount of food sampling and inspection data but has not conducted further mining analysis. At present, a national unified standard food safety index system does not exist [7].

Herein, a new food safety evaluation system was proposed to quantify food safety status as a food safety index, which combines statistical methods and machine learning methods. This model calculates the food safety evaluation index using data on food safety sampling and inspection and other related information. The initial evaluation index was obtained by the optimal distance method, using data on food safety sampling and inspection. Then, expert elicitation via a questionnaire was applied to reweight the results in the four principles of food consumption, project hazards, sales channels, and food production regions. Finally, considering the possible effects of other food safety information on the food safety level, the factor analysis of mixed data method (FADM) was used to modify the reweighted results and obtain the comprehensive food safety evaluation index. The evaluation model constructed in this article creatively introduces FADM weights to minimize the impact of subjective factors on the index calculation. Therefore, the evaluation results reflect the real food safety level. In addition, this calculation model was applied to evaluate the food safety status of a region in China.

This paper has five sections. The Introduction Section outlines the research problem and objectives. The Literature Review Section summarizes and evaluates the relevant existing research. The Data and Methods Section introduces the data selection, sources, and methods applied in this study. The Results and Discussion Section verifies the evaluation model constructed in this paper with actual sampling and inspection data and discusses the results in detail. The Conclusions Section sums up the main points of this study and clarifies the significance of the new findings.

## 2. Literature Review

To meet the demands of food safety management, various approaches to the calculation of the food safety evaluation index have emerged that can be generally categorized into two types as follows: typical statistics-based and machine learning-based. As a widely applied statistics-based method, expert elicitation via a questionnaire is used to collect food safety evaluation results through multiple calls for expert opinions and obtains an evaluation system mainly dependent on experts [8]. To alleviate the subjectivity of expert elicitation, the analytic hierarchy process (AHP) utilizes quantitative and qualitative factor weights to obtain risk levels [8] or criteria weights of food safety by correlation factor analysis [9]. However, data limitation is still a challenge for food safety evaluation. To deal with complex structural data, the fuzzy comprehensive evaluation model (FCEM) is used to quantify an empirical decision to concretize the fuzzy concept and expand information. Moreover, FCEM combined with AHP was proposed to establish the evaluation criteria for evaluation indexes in different dimensions [10]. However, it is still subjective for the above evaluation methods based on statistics. The results are arbitrary, uncertain, and biased because of the inherent properties of these methods, and not all factors can be taken into account.

To obtain objective results, there are evaluation methods based on machine learning for objective data (for example, characteristics description and intake assessment). In studies with limited data, an exposure estimation algorithm was utilized to assess consumer health risks associated with dietary additives [11] and to determine related daily food intake [12]. However, the limited range of evaluation influenced the results [13,14]. As a widely used method in food safety, decision tree evaluation was proposed to deal conservatively with large-scale data, for example, in meat grade classification [15] and the spatial decision tree algorithm for assessing the suitability of garlic [16]. The method produced fuzzy result divisions and incomplete result analysis, which were mainly based on fuzzy features extracted from data [17,18,19]. Considering data from different scales and structures, grey relational analysis (GRA) was used to mine valuable information and analyze the correlation between agricultural product prices and food prices [20] and to reduce identified risks and minimize food waste [21]. GRA is suitable for dynamic analysis, but it also leads to incomplete and non-unique information in the analysis [22]. Moreover, the physiology-based pharmacokinetic model (PBPK) was shown to be an accurate method for large-scale data [23]; it has been used to simulate the pharmacokinetics of compounds under eating and fasting conditions [24] and evaluate the effects of food on the pharmacokinetics of compounds in different biopharmaceutical classification systems (BCSs). Nevertheless, the model requires complex operations and a large amount of data [25,26].

To summarize the above, food safety evaluation often involves various factors and has great differences in data types. It is therefore necessary to develop a comprehensive food evaluation system that incorporates as much useful information as possible to reflect the real food safety level objectively and accurately.

## 3. Data and Methods

### 3.1. Principles for the Evaluation System Construction

To evaluate the overall safety of a specific food, multiple factors were taken into consideration to build a comprehensive and objective evaluation system [27]. This food safety evaluation model comprehensively considers six principles, which can be categorized into quantitative principles (simple qualification degree, food consumption, and project hazard degree) and qualitative principles (sales channels and food production regions).

#### 3.1.1. Basic Index

In this study, a simple qualification degree was selected as the basic index of the comprehensive food safety evaluation model. Different from the qualification ratio (proportion of qualified foods), which is widely used in food safety evaluation, the qualification degree refers to the extent of deviation between the actual test result and the standard value of the food safety limit. Thus, it provides more detailed and reliable evaluation information for food safety management [28].

#### 3.1.2. Food Consumption

The daily consumption of each food was considered in this evaluation system. Ignoring the influence of other factors, the larger the food consumption, the greater the safety risk. For example, in northern regions where grain consumption significantly surpasses that of tea, the negative impact caused by unqualified grain far outweighs that of unqualified tea. Therefore, different food consumption patterns in the evaluation model should elicit corresponding differences in the evaluation results [7,29].

#### 3.1.3. Project Hazards Degree

The toxicological properties of different test projects vary significantly. For example, the consequences of excessive heavy metals in food are far more serious than those of additives. Thus, it is necessary to distinguish the degree of harm from different test projects in the evaluation model. The greater the harm to health, the greater the impact of the test projects should be on the evaluation index [7].

#### 3.1.4. Sales Channels

Food is distributed via various sales channels. There are various sales channels such as supermarkets, fast food restaurants, wholesale markets, snack grocery stores, farm product markets, oversized restaurants, and so on. According to the scale of the channels, the existing sales channels can be categorized into different types. Different types have different effects on food safety. Thus, it is necessary to differentiate among them in the calculation model [29].

#### 3.1.5. Food Production Regions

Regarding food sold in a region, it may be produced locally, sourced from other regions, or even imported from abroad. In the case where there is a safety risk in food produced within a region, the management of that region is fully responsible from production to sales. On the contrary, if the food produced outside the region is a safety risk, the management of that region is only accountable for the sales link. Therefore, in the evaluation model, the significance of locally produced food should be higher than that of food sourced from beyond the region [7,29]. This principle refers specifically to the division of food safety responsibilities under China’s food safety supervision mechanism and can be applied appropriately in other similar situations.

#### 3.1.6. Other Food Safety Sampling and Inspection Information

All the principles mentioned above have a causal relationship with the food safety level. However, food safety is influenced by a lot of factors, and current research has yet to find definitive causality among them. Therefore, it is necessary to incorporate more food safety-related information into the evaluation model and explore potential factors that may affect the food safety level. In this study, machine learning technology was applied to quantify the impact of other food safety factors, such as production and distribution, on the food safety status. 

### 3.2. Data Source

The food safety evaluation system established in this study aims to mine food safety information to reflect the true food safety level and requires different sources and types of data for support. Furthermore, other relevant information and expert opinions were incorporated into the evaluation model to make the calculation results reliable and scientific.

#### 3.2.1. Food Safety Sampling and Inspection Data

The food safety sampling and inspection data used in this study were collected from the National Food Safety Sampling and Inspection Database. Considering the standardization of the sampling and inspection data, this study only selected the data from region X (one of the provinces in China), which contained 15 cities (labeled A, B, C, D, E, F, G, H, I, J, K, L, M, N, and O) in 2020 and 2021, for model construction. The sample consisted of 74,467 unique foods across 8 categories (including dairy products, starch products, grain processing products, meat products, soy products, edible agricultural products, edible oil products, and catering food). 

Based on the characteristics, pollution status, and other factors of each food, 197 test projects with high risk were selected, including 5 categories of heavy metals, mycotoxins, pesticide residues, food additives, and veterinary drug residues (the detailed information is listed in Table S1).

There were 16 sales channels in the original data including vegetable markets, supermarkets, and shopping malls (see Table S2 for details), and food production region information was detailed for the city.

For each unique food sample, 25 valid items were selected from the food safety sampling and inspection data to calculate the FADM weight, including the sampled city, region type, region of labeled production enterprise, and so on (see Table S3 for details). For the convenience of subsequent calculation, the numerical data before screening were processed in a unified and standardized manner [30]. Shelf life was measured in months. The units of the test result, method test limit, and minimum and maximum permissible limits were consistently processed as mg/kg or mg/L to produce data with scientific logic for the following computation.

#### 3.2.2. Food Consumption Data

Food consumption data were collected from the quantity of main commodities purchased per capita by household in the “Statistical Yearbook 2020” of region X. 

#### 3.2.3. Project Hazards Value

The hazard value of test projects was derived according to the hazardous substances scoring principles given by the National Risk Assessment Center.

### 3.3. Construction of Food Safety Evaluation System

In this study, 8 categories and 14 subcategories of the most commonly consumed foods were selected as the food category space (see Table S4 for details).

#### 3.3.1. Simple Qualification Degree

In food safety sampling and inspection, for test item *k* of sample *j*, we assumed the corresponding standard limit value is $M_{jk}$. If $M_{jk}$ is the maximum permissible limit (denoted as ${Max}_{jk}$), it indicates that the greater the test result $T_{jk}$, the less safe the food. If $M_{jk}$ is the minimum permissible limit (denoted as ${Min}_{jk}$), it indicates that the smaller the test result $T_{jk}$, the less safe the food. Some special test items have both a maximum permissible limit ${Max}_{jk}$ and a minimum permissible limit ${Min}_{jk}$. For these types of test items, when the test result $T_{jk}$ is between ${Min}_{jk}\;$and ${Max}_{jk}$, the food is considered safe. However, when $T_{jk}$ is greater than ${Max}_{jk}$ or less than ${Min}_{jk}$, the food is deemed unsafe.

To evaluate the deviation degree of the test results from food safety standard limits, the “simple qualification degree” index Q was designed according to the optimal distance method. The specific calculation method is as follows:

(1) $$Q=\left\{\begin{array}{ll} 0 & T_{jk}\geq {Max}_{jk}\;or\;T_{jk}<{Min}_{jk},\;0\leq {Min}_{jk}{\leq Max}_{jk}\\ 1-\frac{T_{jk}}{{Max}_{jk}} & 0\leq T_{jk}<{Max}_{jk},\;0={Min}_{jk}<{Max}_{jk}\;\\ 1-{\left(\frac{T_{jk}}{{Min}_{jk}}\right)}^{-1} & T_{jk}\geq {Min}_{jk},\;0<{Min}_{jk}={Max}_{jk}\\ 1-\left|\frac{T_{jk}-{Min}_{jk}}{{{Max}_{jk}-Min}_{jk}}-0.5\right| & {Min}_{jk}\leq T_{jk}<{Max}_{jk},\;0<{Min}_{jk}<{Max}_{jk}\\ 1 & T_{jk}=0,\;0={Min}_{jk}={Max}_{jk} \end{array}\right.$$

Q indicates the deviation degree of $T_{jk}$ from $M_{jk},$ and the value ranges from 0 to 1. The closer the Q value is to 1, the smaller the safety risk caused by the corresponding test item of food.

#### 3.3.2. Food Consumption Weight

The original data from the “Statistical Yearbook 2020” were matched with the food categories of food safety sampling and inspection and, finally, the food consumption data applied in this study were obtained. The matching principle is shown in Table S5. 

The calculation formula for the consumption proportion corresponding to different food categories was as follows:

(2) $$\omega_{c}=\frac{n_{i}}{\sum_{k=1}^{n}n_{i}}$$

where $\omega_{c}$ is the food consumption weight and $n_{i}$ is the per capita consumption of food i in region X, for i=1, ⋯, 8.

#### 3.3.3. Expert Elicitation Weight

The weights of project hazards ($\omega_{h})$, sales channels $\left(\omega_{s}\right)$, and food production regions ($\omega_{r}$) were all obtained through expert elicitation via a questionnaire.

The project hazards weight $\omega_{h}$ was calculated based on the hazardous substance scoring table given by the National Risk Assessment Center. The selected 197 test projects with high risk were scored according to standards that combined both acute toxicity and long-term toxicity. The project hazards value ranged from 1 to 5, with 5 points representing the highest risk and 1 point representing the lowest risk (see Table S6 for details).

To calculate the sales channels weight $\omega_{s}$, 16 sales channels were categorized into three types based on their size as follows: large, medium, and small. Through expert elicitation, the weights assigned to large, medium, and small sales channels were 1.0, 1.4, and 1.1, respectively (see Table S7 for details).

For the food production regions weight $\omega_{r}$, foods were similarly categorized into three types as follows: foods produced in the local city, foods produced in region X, and foods produced in other regions. Again, by applying expert elicitation, the weights assigned to foods produced in the local city, region X, and other regions were 1.0, 0.8, and 0.7, respectively (see Table S8 for details).

#### 3.3.4. FADM Weight

In order to mine the internal correlation between food safety status and other food safety data, and to make the evaluation results more objective and comprehensive, the FADM was used in this study to calculate the FADM weigh $\omega_{FADM}$ and quantify this correlation. This part completely relied on machine learning technology in data mining, thus making the results more comprehensive, objective, and accurate. 

A total of *n* samples was selected, each with a total of *p* variables, including *p*_1_ quantitative variables and *p*_2_ qualitative variables. A numerical matrix $X_{\left[1\right]}=\left[X_{\left[1\right]}^{1},\cdots ,X_{\left[1\right]}^{p_{1}}\right]$ composed of *p*_1_ quantitative variables was constructed, and a classification matrix $X_{\left[2\right]}=\left[X_{\left[2\right]}^{1},\cdots ,X_{\left[2\right]}^{p_{2}}\right]$ composed of *p_2_* qualitative variables was also constructed. $X_{\left[1\right]}$ is an $n\times p_{1}$ numerical matrix, while $X_{\left[2\right]}$ is an $n\times p_{2}$ classification matrix. The classification matrix was then transformed into a new matrix of n×m, where *m* represents the total number of levels of *p_2_* qualitative variables.

The specific process was as follows:

(3) $$\left[\begin{array}{cc} \frac{1}{\sqrt{\lambda_{1}^{1}}}X_{\left[1\right]} & \frac{1}{\sqrt{\lambda_{1}^{2}}}X_{\left[2\right]} \end{array}\right]=U\Sigma V^{T}$$

where $\lambda_{1}^{i}$ is the first eigenvalue of matrix $X_{\left[i\right]}$, for i=1, 2. The global matrix is decomposed by singular value decomposition (SVD), where U and V are the left and right singular vectors, respectively, and Σ is the diagonal matrix with singular values on its diagonal.

The matrix F represents the factor scores, and the formula for calculating this matrix is given as follows [31]:

(4) $$F=M^{-\frac{1}{2}}U\Sigma$$

Assuming that each variable factor is of equal importance to the overall model, a diagonal matrix M is constructed. The diagonal elements of M are weights $m_{j}=\frac{1}{n}$, where *n* is the number of factors and $m_{j}\;$represents the weight associated with each factor. In other words, $M=\frac{1}{n}I_{n}$, where $I_{n}$ is the identity matrix of size n×n. Here, the indexes i and j (although not explicitly used in the expression for *M*) typically range from 1 to *n*.

Because the expression of factor scores are deviations from the mean, factor scores must be converted in such a way that their minimum value becomes zero before they can be calculated as indicators. The formula is as follows [5]:

(5) $$f\left(F_{i}\right)=\left\{\begin{array}{ll} 1+\frac{k-1}{2}e^{F_{i}} & F_{i}\geq 0\\ k-\frac{k-1}{2}e^{{-F}_{i}} & F_{i}<0 \end{array}\right.$$

where the parameter k represents the asymptotic degree of the control transformation and is the range value of the transformation factor value. Therefore, if k=100, it indicates that the transformation will generate a value with 100 as the upper limit. 

Finally, the modified factor weight formula is obtained by the FADM as follows:

(6) $$\omega_{FADM}=\sum_{i=1}^{n}f\left(F_{i}\right)\times \frac{\omega_{i}}{\sum_{i=1}^{n}\omega_{i}}$$

where i is the number of the last principal factor, $F_{i}$ is the value of the i principal factor, $f\left(F_{i}\right)$ is the factor score after conversion, and $\omega_{i}$ is the variance contribution rate of the i principal factor. 

#### 3.3.5. Food Safety Evaluation Index System

Considering all the factors such as a simple qualification degree, food consumption, project hazards, sales channels, food production regions, and the FADM correction factor, the calculation formula for the comprehensive food safety qualification degree $\bar{Q}$ was obtained as follows:

(7) $$\bar{Q}={\omega_{c}\cdot \omega }_{h}\cdot \omega_{s}\cdot \omega_{r}\cdot \omega_{FADM}\cdot Q$$

where $\omega_{c}$ is the food consumption weight, $\omega_{h}$ is the project hazards weight ($a_{i}=1,\;\cdots ,5$), $\omega_{s}$ is the sales channels weight ($\omega_{s=large}=1.0,\omega_{s=medium}=1.4,\omega_{s=small}=1.1$), $\omega_{r}$ is the food production region’s weight ($\omega_{r=local\;city}=1.0,\omega_{r=local\;region}=0.8,\omega_{r=other\;region}=0.7$), $\omega_{FADM}$ is the FADM correction factor weight, and Q is the simple qualification degree. 

The calculation formula for city t’s comprehensive food safety qualification degree ${\bar{R}}^{\left(t\right)}$ is as follows:

(8) $${\bar{R}}^{\left(t\right)}=\frac{1}{n}\sum_{i=1}^{n}\bar{Q_{i}}$$

where n is the number of samples in city t and $\bar{Q_{i}}$ is the comprehensive food safety qualification degree of sample *j*.

The calculation formula for food category i’s comprehensive food safety qualification degree ${\bar{R}}^{\left(i\right)}$ is as follows:

(9) $${\bar{R}}^{\left(i\right)}=\frac{1}{n}\sum_{i=1}^{n}\bar{Q_{i}}$$

where n is the number of samples in food category i and $\bar{Q_{i}}$ is the comprehensive food safety qualification degree of sample *j*.

**Figure 1:**
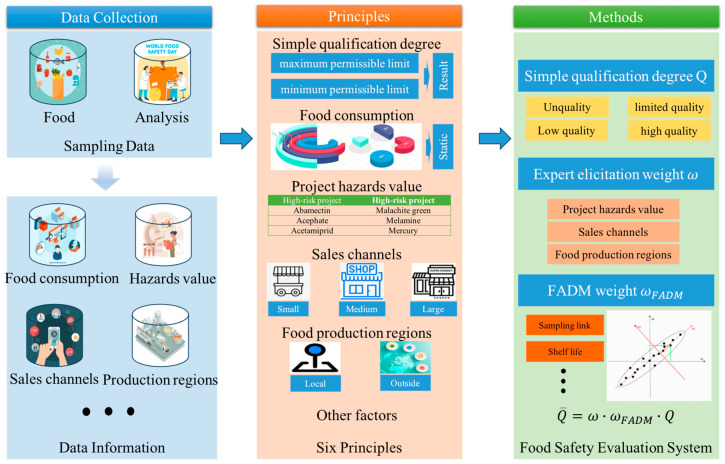
The framework of the food safety evaluation system.

Overall, the framework of the food safety evaluation system is shown in Figure 1.

#### 3.3.6. Verification of the FADM 

Difference indicators are often used to analyze the difference in the results between an experimental and the control group and whether this difference is significant. The difference between the two results is expressed by *p*. *p* < 0.05 indicates that the difference is significant and, vice versa, indicating that the difference is not significant. Since expert elicitation via a questionnaire is commonly used for food safety evaluation, in order to show the characteristics of the FADM, we applied comparative experiments of the two methods and analyzed the results by the difference index *p*. In this article, the FADM-based food evaluation index method and expert elicitation are compared, so the test results of the paired sample *t* are selected as the *p* value for analysis. The formula is as follows:

(10) $$t=\frac{\bar{X_{diff}}}{\frac{S_{diff}}{\sqrt{n}}}$$

where $\bar{X_{diff}}$ is the mean value of the difference between the two method results, $S_{diff}$ is the standard deviation of the difference, and n is the sample size.

## 4. Results and Discussion

According to the food safety evaluation model constructed in this paper, the food safety status of region X was calculated and analyzed. Table 1 shows that the annual average of the food safety index in region X obtained by the FADM fluctuated between 50 and 70 in two years. Compared with expert elicitation, the average food safety indexes of sampled city and food category by the FADM were very similar (66.6 and 64.4 in 2020, 55.8 and 50.4 in 2021), and the food safety indexes had obvious changes in different years that can be clearly observed. Therefore, the overall food safety situation in region X was stable and controllable in these two years, which was consistent with the food safety situation in China as a whole. Moreover, the evaluation system constructed here can accurately reflect the overall level of food safety in a region.

**Figure 2:**
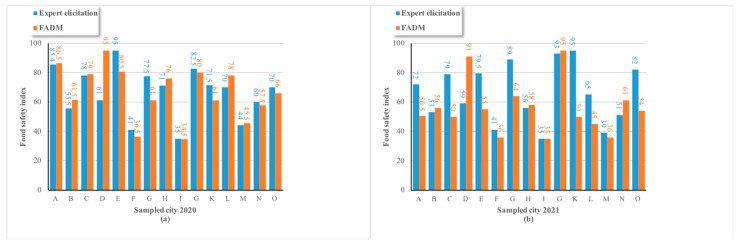
The food safety indexes of 15 cities were calculated using expert elicitation and the FADM. (**a**) Food safety indexes of 15 cities in region X in 2020. (**b**)Food safety indexes of 15 cities in region X in 2021.

In detail, Figure 2 shows the food safety indexes of 15 cities in region X. Among the food safety indexes of the 15 cities in 2020, the results by the weight method based on the FADM showed that city D was the highest (95), and the top three cities were D, A, and E, while city I was the lowest (35). Similarly, in 2021, the results indicated that city J was the highest (95), and the top three cities were J, D, and G, with city I remaining the lowest (35). Comparing the two years, the distribution of the food safety index across cities was inconsistent, yet two of the top three cities repeated in both years (A and E in 2020, G and J in 2021), and city I consistently had the lowest food safety index. Furthermore, city D maintained a high level of the food safety index in both years (first and second, respectively), highlighting its relatively good food safety status compared with the other cities in region X, whereas city I consistently ranked the lowest. 

Figure 2a shows a comparison of the two methods on the sampled cities in 2020. A *p* value of 0.99 was obtained by calculating the difference index. Figure 2b shows a comparison of the two methods in 2021, and a *p* value of 0.15 was obtained. From the perspective of the sampled cities, the *p* value is greater than 0.05 in both years. Therefore, the difference between the two methods is not significant, indicating that the FADM method can be used to calculate food safety evaluation indexes. More objective results can be obtained by utilizing data feature information.

Also, different from expert elicitation, the FADM can obviously reflect changes in the food safety level of different cities in different years, which makes it possible to analyze the food safety change trend in a particular city. A possible reason is that expert elicitation only considers the simple qualification degree and expert weight in different cities, but the FADM comprehensively incorporates more food safety-related factors into the evaluation model to make the results more objective and comprehensive.

**Figure 3:**
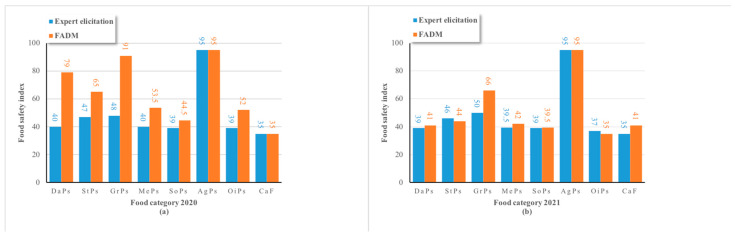
The food safety indexes of eight food categories were calculated using expert elicitation and the FADM. (**a**) Food safety indexes of eight food categories in region X in 2020. (**b**) Food safety indexes of eight food categories in region X in 2021. Abbreviations: DaPs—dairy products, StPs—starch products, GrPs—grain processing products, MePs—meat products, SoPs—soy products, AgPs—edible agricultural products, OiPs—edible oils products, CaF—catering food.

Figure 3 shows the food safety indexes of eight food categories in region X. Among the food safety indexes of the eight food categories in both 2020 and 2021, the FADM-based weight method showed that the top two food categories were edible agricultural products (95 in both years) and grain processing products (91 in 2020 and 66 in 2021). The reason is that agricultural products and grain products are the two food categories with the largest national consumption in China, and supervision and financial investment are relatively higher. However, the food safety indexes of edible oil products and catering food were low in both years, reflecting the high safety risk of these two food categories in region X. Daily supervision and sampling monitoring should be strengthened. 

Figure 3a shows a comparison of two methods for food categories in 2020. A *p* value of 0.77 was obtained by calculating the difference index. Figure 3b shows a comparison of the two methods in 2021, where *p =* 0.13 was obtained. From the perspective of food categories, the *p* value is greater than 0.05 in both years, indicating that the difference between the two methods is not obvious. However, the evaluation results obtained by the FADM vary greatly between the two years, while the results of expert elicitation remained largely unchanged. This is mainly due to the fact that the FADM incorporates information on food categories, sampled cities, and sampling links. As a result of more data and information, the method can express the evaluation results more comprehensively and accurately.

In addition, the FADM produced greatly varying results across different food categories in both years, whereas the expert elicitation approach failed to clearly distinguish the safety levels of the remaining seven food categories, excluding edible agricultural products. This is primarily because the FADM model incorporates a wider range of food safety-related factors, enabling it to reflect the actual food safety status accurately and objectively.

Because of the limited data accumulation (only two years of data), the accuracy and comprehensiveness of the evaluation results are affected. Therefore, it is necessary to enrich the sampling data to optimize the evaluation model, with the aim of improving the reliability of the evaluation index and verifying the generalizability of this mothed. In order to further enhance the accuracy of food safety evaluation results, consumption differences in different cities should be taken into account during sampling, and the number and categories of samples should be reasonably selected to obtain data that can objectively reflect the real food safety level.

## 5. Conclusions

In this paper, a novel calculation method for food safety evaluation indexes is proposed, which combines statistical and machine learning methods. Based on optimal value distance and expert elicitation weights, the FADM is applied to refine the evaluation results, ensuring objectivity and comprehensiveness of the data. This method is then applied to analyze the food safety level of region X from 2020 to 2021, examining both sampled cities and food categories. The following conclusions are drawn:

From an evaluation standpoint, the FADM-based food safety evaluation system presented in this paper accurately, objectively, and comprehensively reflects the food safety status. The results can be analyzed from multiple angles to cater to varying evaluation requirements. In particular, during application analysis, regional differences in food sales and dietary patterns must be taken into consideration, leading to variations in the weights assigned to different foods and detection indicators when calculating the food safety evaluation index.

From a supervision perspective, the calculation model constructed in this paper employs the food safety index to quantify the food safety situation, enabling us to gain an intuitive understanding of the food safety level. This model can identify cities and food categories with heightened food safety risks, thereby facilitating the rational allocation of regulatory resources and providing technical and theoretical support for government decision-making.

The implementation of food safety evaluation in region X demonstrates that the model developed in this paper is both effective and easily adaptable for widespread use. However, there are some inadequacies, such as insufficient data. In the subsequent stage, we plan to grade the food safety level based on the magnitude of the evaluation index to prevent the undue pursuit of a high safety index, ensuring that the evaluation more accurately reflects the regional food safety situation.

## Figures and Tables

**Table 1:** The annual average of the food safety index in region X.

Year	Classification	Expert Elicitation	FADM
2020	Sampled city	66.5	66.6
	Food category	47.9	64.4
2021	Sampled city	65.9	55.8
	Food category	47.6	50.4

## Data Availability

The original contributions presented in the study are included in the article/supplementary material, further inquiries can be directed to the corresponding authors.

## Supplementary Materials

The following supporting information can be downloaded at https://www.mdpi.com/article/10.3390/foods13172680/s1, Table S1. A total of 197 high-risk projects identified based on the characteristics of each food, pollution status of food pollutants, and other factors. Table S2. The 16 sales channels represented in the food sampling data. Table S3. Selection of 25 valid items based on food sampling data. Table S4. Eight categories with 14 subcategories of commonly used foods. Table S5. The matching principle for food categories. Table S6. Evaluation of hazard value a_j_ of test projects using the acute and long-term toxicity of hazardous substances. Table S7. The weighting of sales channels determined by expert elicitation. Table S8. The weighting of food production regions determined by expert elicitation. Table S9. Algorithm flow of the food safety evaluation system. Figure S1. Data equilibrium distribution.
