# Supplementary material for: Construction of a Food Safety Evaluation System Based on the Factor Analysis of Mixed Data Method
Source: Foods. 2024 Aug 25;13(17):2680. doi: 10.3390/foods13172680 (PMC11394990; doi:10.3390/foods13172680)
Supplement: Supplementary file 1 [file foods-13-02680-s001.zip › foods-3106314-supplementary.pdf]

Supplementary Materials

Table S1. 197 high-risk projects identified based on characteristics of each food, pollution status of food pollutants, and other factors.

Number	High-risk projects	Number	High-risk projects
**1**	6-benzylaminopurine	**100**	Melophos
**2**	Abamectin	**101**	Mercury
**3**	Acephate	**102**	Metabolites of furotadone
**4**	Acesulfame Potassium	**103**	Metalaxyl and fine metalaxyl
**5**	Acetamiprid	**104**	Methamidophos
**6**	Acid Orange II	**105**	Methanol
**7**	Acid value	**106**	Methomyl
**8**	Acid value (KOH)	**107**	Methyl parathion
**9**	Acidity	**108**	Methylmercury
**10**	Aflatoxin B1	**109**	Methylthiocyclophosphorus
**11**	Aflatoxin M_1	**110**	Metophos
**12**	Aldicarb	**111**	Metronidazole
**13**	Aluminium	**112**	Morphine
**14**	Amantadine	**113**	Mould
**15**	Amaranth red	**114**	Nacodine
**16**	Amino acid nitrogen	**115**	Natamycin
**17**	Ammonium salt	**116**	Nicarbazine
**18**	Anionic synthetic detergent	**117**	Nitrile benzozole
**19**	Arsenic	**118**	Nitrite
**20**	Aspartame	**119**	N-nitrosod imethylamine
**21**	Aureomycin	**120**	Non-fat milk solids
**22**	Barium	**121**	Norfloxacin
**23**	Benzoapyrene,	**122**	Number of lactic acid bacteria
**24**	Benzoic acid	**123**	Ofloxacin
**25**	Benzoyl peroxide	**124**	Olaquindox
**26**	Bifenthrin	**125**	Omethoate
**27**	Borax	**126**	Oxytetracycline
**28**	Bright blue	**127**	Papaverine
**29**	Bromate	**128**	Parathion
**30**	Butyl hydroxyanisole (BHA)	**129**	Pefloxacin
**31**	Cadmium	**130**	Pentachlorophenol sodium
**32**	Carbendazim	**131**	Peroxide value
**33**	Carbofuran	**132**	Phorate
**34**	Carmine	**133**	Phoxim
**35**	Chloramphenicol	**134**	P-hydroxy benzoates and its salts
**36**	Chlorothalonil	**135**	Polychlorinated biphenyls
**37**	Chlorpromazine	**136**	Potassium acetylsulfonate
**38**	Chlorpyrifos	**137**	Potassium ferrocyanide
**39**	Chromium	**138**	Prochloraz and prochloraz

Number	High-risk projects	Number	High-risk projects
			manganese salt
**40**	Clenbuterol	**139**	Profenofos
**41**	Codeine	**140**	Propanediol
**42**	Coliform group	**141**	Propionic acid
**43**	Commercial sterility	**142**	Protein
**44**	Cyanide	**143**	Pseudomonas aeruginosa
**45**	Cyclamate	**144**	Pyraclostrobin
**46**	Cyfluthrin and cyfluthrincyhalothrin	**145**	Pythium
**47**	Cyfluthrin and cypermethrin	**146**	Ractopamine
**48**	Cypermethrin and beta-cypermethrin	**147**	Residual chlorine
**49**	Cyromazine	**148**	Rhodamine B
**50**	Dafloxacin	**149**	Ribavirin
**51**	DDT	**150**	Saccharin sodium
**52**	Dehydroacetic acid and its sodium salt	**151**	Salbutamol
**53**	Deltamethrin	**152**	Salmonella
**54**	Deoxynivalenol	**153**	Selenium
**55**	Dexamethasone	**154**	HCH
**56**	Diazepam	**155**	Sodium
**57**	Dibutyl hydroxytoluene (BHT)	**156**	Sodium 4-chlorophenoxyacetate
**58**	DicofolTrichlorfon	**157**	Sodium chloride
**59**	Dietary fiber	**158**	Sodium formaldehyde bisulfite
**60**	Difenoconazole	**159**	Sorbic acid and its potassium salt
**61**	Diflubenzuron	**160**	Staphylococcus aureus
**62**	Dimethoate	**161**	Starch
**63**	Dimethyl fumarate	**162**	Sucralose
**64**	Dimethylpentyl	**163**	Sucrose
**65**	Disodium ethylenediamine tetraacetic acid	**164**	Sudan I
**66**	Doxycycline	**165**	Sudan II
**67**	Endosulfan	**166**	Sudan III
**68**	Enoylmorpholine	**167**	Sudan IV
**69**	Enrofloxacin	**168**	Sulfite
**70**	Escherichia coli	**169**	Sulfonamides
**71**	Ethyl maltol	**170**	Sulfur dioxide residue
**72**	Fat	**171**	Sunset yellow
**73**	Fenitrothion	**172**	Talc
**74**	Fenthion	**173**	TBHQ
**75**	Fenvalerate and S-fenvalerate	**174**	Tebuconazole
**76**	Fipronil	**175**	Terbutaline
**77**	Florfenicol	**176**	Tetracycline

Number	High-risk projects	Number	High-risk projects
**78**	Fluorescent substance	**177**	Thiabendazole
**79**	Fluoromethylquine	**178**	Tibain
**80**	Forchlorfenuron	**179**	Titania
**81**	Free mineral acid	**180**	Total acid
**82**	Free residual chlorine	**181**	Total bacterial count
**83**	Fructose and glucose	**182**	Triazophos
**84**	Furacilin metabolite	**183**	Trichlorfon
**85**	Furantoin metabolite	**184**	Trichloromethane
**86**	Furazolidone metabolite	**185**	Trimethoprim
**87**	Herceptin A	**186**	Turbidity
**88**	Imidacloprid	**187**	Volatile basic nitrogen
**89**	Iodine	**188**	Water content
**90**	Isazofos	**189**	Zearalenone
**91**	Isocarbophos	**190**	Zinc
**92**	Isophos methyl	**191**	Metasilicic acid
**93**	Lead	**192**	Chlordimeform
**94**	Lemon yellow	**193**	Dichlorvos
**95**	Listeria monocytogenes	**194**	Yeast
**96**	Strontium	**195**	Carbamectin benzoate
**97**	Lomefloxacin	**196**	Ochratoxin A
**98**	Malachite green	**197**	Lactobacillus
**99**	Melamine		

The 197 high-risk projects encompass various types, including heavy metals, mycotoxins, pesticide residues, food additives and veterinary drug residues.

Table S2. 16 sales channels represented in food sampling data.

Number	Sampled area	Sales channels
1	Raw and auxiliary material warehouse	Large
2	Finished product warehouse (inspected area)	Large
3	Wholesale market	Large
4	Oversized restaurant	Large
5	Group meal delivery unit	Large
6	Farm product market	Medium
7	Market	Medium
8	School/kindergarten canteen	Medium
9	Food market	Medium
10	Supermarket	Medium
11	Midsize restaurant	Medium
12	Other	Small
13	Snack bar	Small
14	Small restaurant	Small
15	Snack grocery store	Small
16	Fast food restaurant	Small

Table S3. Selection of 25 valid items based on food sampling data.

Number	Items	Number	Items
1	Sampled city	14	Sub-food category
2	Region type	15	Food species
3	Region of labeled production enterprise	16	Specific food category
4	Sample name	17	Sample type
5	Update time	18	Package classification
6	Sample number	19	Test item
7	Sample quantity	20	Test result
8	Sampling process	21	Result judgment
9	Whether to import	22	Method test limit
10	Sample specification	23	Minimum permissible limit
11	Sampling site	24	Maximum permissible limit
12	Shelf life	25	Test conclusion
13	Food category		

Table S4. 8 categories with 14 subcategories of commonly used foods.

Number	Food category	Number	Sub-food subcategory
**1**	Grain processing products	**1**	Wheatflour
		**2**	Rice
**2**	Edible oil products	**3**	Edible vegetable oil
**3**	Catering food	**4**	Catering food
**4**	Soy products	**5**	Soy products
**5**	Edible agricultural products	**6**	Vegetable
		**7**	Livestock and poultry meat and by-products
		**8**	Aquatic products
		**9**	Eggs
		**10**	Fruits
		**11**	Raw and dried nuts and seeds
**6**	Dairy products	**12**	Dairy products
**7**	Starch products	**13**	Starch products
**8**	Meat products	**14**	Cooked meat products

Table S5. The matching principle for food categories.

Number	Statistical Yearbook	Food Safety Sampling And Inspection	
		Sub-food category	Food category
1	Wheat<br>Flour	Wheatflourl	Grain processing products
	Rice	Rice	
2	Edible vegetable oil	Edible vegetable oil	Edible oil products
3	Pastry food	Catering food	Catering food
4	Beans	Soy products	Soy products
5	Vegetable	Vegetable	Edible agricultural products
	Pork<br>Beef<br>Mutton<br>Chicken<br>Duck	Livestock and poultry meat and by-products	
	Fish<br>Shrimps	Aquatic products	
	Eggs	Eggs	
	Fruits	Fruits	
	Raw and dried nuts and seeds	Raw and dried nuts and seeds	
6	Milk<br>Yogurt<br>Milk powder	Dairy products	Dairy products
7	Potato	Starch products	Starch products
8	Pork<br>Beef<br>Mutton<br>Chicken<br>Duck	Cooked meat products	Meat products

Table S6. Evaluation of hazard value a_j of test projects using acute and long-term toxicity of hazardous substances.

Assignment	Harm	
	Acute toxicity	Long-term toxicity
	Rat oral LD_{50}(mg/kg bw)	ADI/ TDI/PTWI/PTMI
1	> 5000	Hazardous substances with ADI or TDI>10 μg/kg bw day; For substances with no ADI or TDI value, the hazards include: Carcinogens (category 4); neurotoxicity (category 4); uncertain health hazard effects and unclassified toxic or harmful substances; substances not classified as toxic or harmful.
2	$501 - 5000$	Hazardous substances with ADI or TDI =0.1-10 μg/kg bw day; For substances with no ADI or TDI value, the hazards include: Carcinogens (category 3); neurotoxicity (category 3).
3	$51 - 500$	Hazardous substances with ADI or TDI =0.001-0.1 μg/kg bw day; For substances with no ADI or TDI value, the hazards include: Carcinogens (category 2B); neurotoxicity (category 2); mutagens (category 3); reproductive / developmental toxicity (category 3); chronic toxicity (category 2).
4	$1 - 50$	Hazardous substances with ADI or TDI <0.001 μg/kg bw day; For substances with no ADI or TDI value, the hazards include: Carcinogens (category 2A); neurotoxicity (category 1); mutagens (category 2); reproductive / developmental toxicity (category 2); chronic toxicity (category 1).
5	< 1	Hazardous substances with ADI or TDI <0.001 μg/kg bw day; For substances with no ADI or TDI value, the hazards include: Carcinogens (category 1); mutagens (category 1); reproductive / developmental toxicity (category 1).

LD_{50} refers to the half lethal dose of oral exposure; ADI: Acceptable Daily Intake, it refers to the dose of certain chemical substances (food additives, pesticides, etc.) consumed by the end person or animal every day without any known adverse effects on health; TDI: Tolerable Daily Intake, it refers to the allowable amount of lifetime intake of a substance without an estimated risk of harmful health, and it is the safety limit for the intake of a substance.

Table S7. Weighting of sales channels determined by expert elicitation.

Number	Sales channels		
	Large	Medium	Small
**Expert 1**	1.0	0.5	0.5
**Expert 2**	1.0	3.0	1.0
**Expert 3**	1.0	1.0	0.5
**Expert 4**	1.0	1.0	1.0
**Expert 5**	1.0	1.0	0.8
**Expert 6**	1.0	0.9	0.8
**Expert 7**	1.0	2.0	1.0
**Expert 8**	1.0	0.8	0.6
**Expert 9**	1.0	1.0	1.0
**Expert 10**	1.0	0.5	0.3
**Expert 11**	1.0	0.8	0.5
**Expert 12**	1.0	2.0	4.0
**Expert 13**	1.0	0.8	0.6
**Expert 14**	1.0	2.0	2.0
**Expert 15**	1.0	3.0	2.0
**mean value**	**1.0**	**1.4**	**1.1**
**median**	1.0	1.0	0.8
**standard deviation**	0.0	0.8	0.9
**range**	0.0	2.5	3.8

Table S8. Weighting of food production regions determined by expert elicitation.

Number	Food production regions		
	Local city	Region X	Other regions
**Expert 1**	1.0	0.6	0.3
**Expert 2**	1.0	0.5	0.5
**Expert 3**	1.0	1.0	1.0
**Expert 4**	1.0	0.6	0.6
**Expert 5**	1.0	0.5	0.5
**Expert 6**	1.0	0.7	0.6
**Expert 7**	1.0	1.0	0.5
**Expert 8**	1.0	0.8	0.6
**Expert 9**	1.0	0.8	0.6
**Expert 10**	1.0	0.8	0.4
**Expert 11**	1.0	1.0	1.0
**Expert 12**	1.0	0.8	0.8
**Expert 13**	1.0	1.0	1.0
**Expert 14**	1.0	1.0	1.0
**Expert 15**	1.0	0.3	1.0
**mean value**	**1.0**	**0.8**	**0.7**
**median**	1.0	0.8	0.6
**standard deviation**	0.0	0.2	0.2
**range**	0.0	0.7	0.7

Data Normalization Processing:

For the convenience of subsequent data calculation, the numerical data before screening are uniformly normalized. The shelf life time is processed in months. If the shelf life is two years, it is recoded as 24 months; if the shelf life is 15 days, it is recorded as 0.5 months. The units of the test results, the detection limit, the minimum permissible limit, and the maximum permissible limit are unified to provide scientific and reasonable data for subsequent evaluation. The qualitative data are normalized, and the formula is as follows:

$$x = \frac{X_{max} - X}{X_{max} - X_{min}}$$

Where, x is the normalized data, X is the original data, X_{max} is the maximum value in the original data, and X_{min} is the minimum value in the original data.

Missing values and Outliers Processing:

Because the sampling data is collected and entered manually, data missing is inevitable. For the data missing value, we apply the mean supplement method to fill the data. Because of the existence of 'dirty data' in the sampling data, which can also be called 'outliers', these data will cause the deviation of the overall characteristics of the data. In order to find out the outliers, we directly delete them.

Data Equilibrium:

When calculating the simple qualification degree, the differences between the data cannot be clearly distinguished because the calculation results are too concentrated in the range of (0.8, 1.0). Therefore, it is not conducive to accurate calculation and clear results display. This paper uses the

frequency histogram method to make the data balanced, so that the data distribution in the interval is more even, and the results are shown in Figure S1:

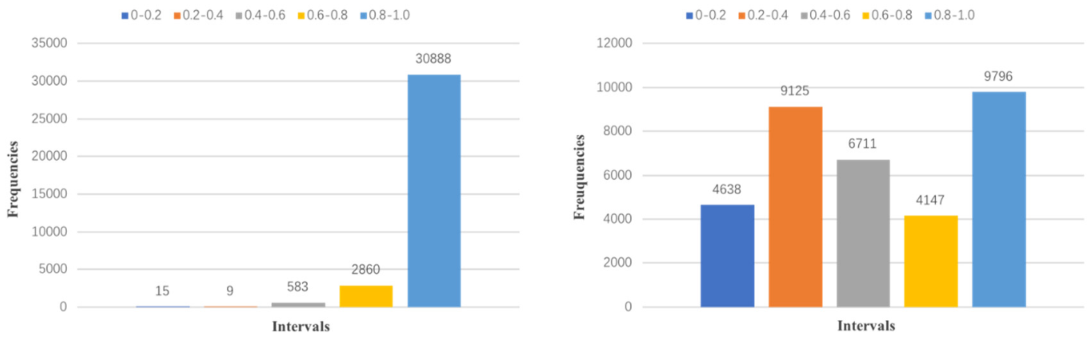

Figure S1. Data Equilibrium Distribution

Table S9. Algorithm Flow of Food Safety Evaluation System

**Algorithm: Food Safety Evaluation System Based on FADM**
**Input:** Food sampling data set $F = \{(x^{(1)}, y^{(1)} \cdots), (x^{(2)}, y^{(2)} \cdots), \cdots (x^{(m)}, y^{(m)} \cdots)\}$
**Output:** Comprehensive food safety qualification $\bar{Q}$
Normalization: F
Calculate simple qualification Q
**if** $0 = Min_{jk} = Max_{jk}$ **then**
**if** $T_{jk} = 0$ **then** $Q = 1$ **else** $Q = 0$
**else if** $0 = Min_{jk} < Max_{jk}$ **then**
**if** $0 \leq T_{jk} < Max_{jk}$ **then** $Q = 1 - \frac{T_{jk}}{Max_{jk}}$ **else** $Q = 0$
**else if** $0 < Min_{jk} = Max_{jk}$ **then**
**if** $T_{jk} \geq Min_{jk}$ **then** $Q = 1 - \left(\frac{T_{jk}}{Min_{jk}}\right)^{-1}$ **else** $Q = 0$
**else** $0 < Min_{jk} = Max_{jk}$ **then**
**if** $Min_{jk} \leq T_{jk} < Max_{jk}$ **then** $Q = 1 - \left\lvert\frac{T_{jk} - Min_{jk}}{Max_{jk} - Min_{jk}} - 0.5\right\rvert$ **else** $Q = 0$
**end**
Calculate expert elicitation weight $\omega_c, \omega_h, \omega_s, \omega_r$
Calculate FADM weight $\omega_{FADM} = \sum_{i=1}^{n} f(F_i) \times \frac{\omega_i}{\sum_{i=1}^{n} \omega_i}$
Food safety evaluation index system $\bar{Q} = \omega_c \cdot \omega_h \cdot \omega_s \cdot \omega_r \cdot \omega_{FADM} \cdot Q$
